# Tuning the Electronic Structure of LaNiO_3_ through Alloying with Strontium to Enhance Oxygen Evolution Activity

**DOI:** 10.1002/advs.201901073

**Published:** 2019-08-07

**Authors:** Jishan Liu, Endong Jia, Le Wang, Kelsey A. Stoerzinger, Hua Zhou, Chi Sin Tang, Xinmao Yin, Xu He, Eric Bousquet, Mark E. Bowden, Andrew T. S. Wee, Scott A. Chambers, Yingge Du

**Affiliations:** ^1^ State Key Laboratory of Functional Materials for Informatics Shanghai Institute of Microsystem and Information Technology Chinese Academy of Sciences Shanghai 200050 China; ^2^ Center for Excellence in Superconducting Electronics Chinese Academy of Sciences Shanghai 200050 China; ^3^ Physical and Computational Sciences Directorate Pacific Northwest National Laboratory Richland WA 99354 USA; ^4^ The Key Laboratory of Solar Thermal Energy and Photovoltaic System Institute of Electrical Engineering Chinese Academy of Sciences Beijing 100190 China; ^5^ Department of Physics University of Chinese Academy of Sciences Beijing 100190 China; ^6^ School of Chemical Biological and Environmental Engineering Oregon State University Corvallis OR 97331 USA; ^7^ X‐Ray Science Division Advanced Photon Source Argonne National Laboratory Lemont IL 60439 USA; ^8^ Department of Physics Faculty of Science National University of Singapore Singapore 117542 Singapore; ^9^ NUS Graduate School for Integrative Sciences and Engineering National University of Singapore Singapore 117456 Singapore; ^10^ Theoretical Materials Physics Q‐MAT Cesam University of Liège B‐4000 Liège Belgium; ^11^ Environmental Molecular Sciences Laboratory Pacific Northwest National Laboratory Richland WA 99354 USA

**Keywords:** hole doping, hybridization, LaNiO_3_, nickelates, oxygen evolution reaction

## Abstract

The perovskite oxide LaNiO_3_ is a promising oxygen electrocatalyst for renewable energy storage and conversion technologies. Here, it is shown that strontium substitution for lanthanum in coherently strained, epitaxial LaNiO_3_ films (La_1−_ *_x_*Sr*_x_*NiO_3_) significantly enhances the oxygen evolution reaction (OER) activity, resulting in performance at *x* = 0.5 comparable to the state‐of‐the‐art catalyst Ba_0.5_Sr_0.5_Co_0.8_Fe_0.2_O_3−_ *_δ_*. By combining X‐ray photoemission and X‐ray absorption spectroscopies with density functional theory, it is shown that an upward energy shift of the O 2p band relative to the Fermi level occurs with increasing *x* in La_1−_ *_x_*Sr*_x_*NiO_3_. This alloying step strengthens Ni 3d–O 2p hybridization and decreases the charge transfer energy, which in turn accounts for the enhanced OER activity.

The electrolytic decomposition of earth‐abundant H_2_O, consisting of the oxygen evolution and hydrogen evolution half‐cell reactions, holds significant promise for generating clean H_2_ fuel.^1^ However, the slow kinetics of the oxygen evolution reaction (OER) limits the efficiency of the overall process. ABO_3_‐type perovskite oxides are attractive electrocatalysts for promoting the OER in alkaline environments,^2^, ^3^, ^4^ in that their activities are comparable to those of benchmark catalysts such as RuO_2_ and IrO_2_, both of which employ expensive and scarce metals.^3^

The OER activity of perovskite oxides is strongly dependent on their electronic structure, and many studies have focused on identifying activity descriptors based on the electronic properties of the surface or bulk.^3^, ^4^, ^5^, ^6^, ^7^, ^8^ These descriptors span from molecular orbital based models to band theory and include the number of d electrons,^9^ e_g_ orbital filling,^3^, ^10^ metal 3d–oxygen 2p hybridization,^11^ the position of the oxygen p band center relative to the Fermi level (*E*_F_),^2^, ^12^ and charge transfer energy (*∆*).^5^ Some studies suggest that the chemical properties of perovskite oxides are controlled by the metal 3d band filling.^2^, ^13^ Previous calculations show that a lower metal 3d band center weakens the metal–oxygen chemisorption energy,^13^ which is believed to be favorable for enhanced OER activity.^2^ Others have taken a more localized approach, focusing on e_g_ orbital filling in the B‐site transition metal cation. In this paradigm, an e_g_ occupancy slightly greater than unity is considered optimal for OER.^3^ However, in comparing materials with similar e_g_ occupancies, significant differences in OER activities still exist,^3^, ^14^ underscoring the importance of considering the nature of metal–oxygen orbital overlap in descriptions of oxygen electrocatalysis. Recently, the charge transfer energy *∆*, which is the energy difference between the oxygen 2p and metal 3d orbital energies, was proposed as a key electronic descriptor for OER activity.^5^

Perovskite‐structured nickelates, in particular LaNiO_3_ (LNO), have attracted considerable attention for their desirable OER performance.^3^ Various approaches including strain engineering,^2^, ^15^ cation nonstoichiometry controlling^16^ have been explored in pursuit of enhancing the OER activity of nickelates. Aliovalent substitution of Sr^2+^ or Ba^2+^ for A‐site La^3+^ in LaMO_3_ (M = Co, Fe),^6^, ^7^, ^17^, ^18^, ^19^ has been demonstrated to be an effective approach for enhancing OER activity. Recently, Sankannavar et al.^20^ reported that Sr‐doped LNO powders show significantly higher OER activity than pure LNO. However, according to the e_g_ occupancy design principle,^3^ La_1−_ *_x_*Sr*_x_*NiO_3_ (LSNO) formation would essentially reduce the e_g_ orbital occupancy to less than unity, which can be unfavorable for OER.^2^, ^3^ It is thus important to examine this discrepancy to elucidate how electronic structure and OER activity of La_1−_ *_x_*Sr*_x_*NiO_3_ evolve as a function of *x*.

We have synthesized a set of compositionally and structurally well‐defined epitaxial LSNO films over the full range of compositions and have investigated the resulting properties to establish the impact of Sr alloying on LSNO electronic structure and OER activity. The films were deposited using oxygen plasma–assisted molecular beam epitaxy (OPA‐MBE). In contrast to previous studies of LSNO particles,^20^ these epitaxial films have controlled surface areas and orientations, and are free of secondary phases such as NiO and SrCO_3_. These films are thus ideally suited for fundamental investigations to reveal the underlying mechanisms. In‐plane transport measurements confirm that the LSNO films become insulating when *x* exceeds 0.5 (Figure S1, Supporting Information). Due to the large potential voltage drop across the films, we cannot reliably measure OER activity for La_0.25_Sr_0.75_NiO_3_ and SrNiO_3−_ *_δ_*. However, within the measurable range (0 ≤ *x* ≤ 0.5), the OER activity is found to increase with *x*, with *x* = 0.5 being the most active. By combining X‐ray photoemission spectroscopy (XPS) and X‐ray absorption spectroscopy (XAS) with density functional theory (DFT) calculations, we show that increasing the Sr mole fraction in LSNO system induces an upward shift of the O 2p bands relative to the Fermi level, strengthening Ni 3d–O 2p hybridization, and decreasing *∆*, which accounts for the enhanced OER activity.

Epitaxial LSNO films of thickness equal to 20 unit cells (u.c.) were grown on (001)‐oriented LaAlO_3_ (LAO) substrates by OPA‐MBE.^21^, ^22^ Bulk LNO exhibits a rhombohedral structure with a room‐temperature pseudocubic lattice constant of ≈3.84 Å.^23^ LAO with its pseudocubic lattice constant of ≈3.794 Å was used as the substrate because of its small in‐plane lattice mismatch with LNO. **Figure** **1**a shows a schematic illustration of the alternating deposition and annealing approach to grow these LSNO films. The elemental fluxes for La, Sr, and Ni were calibrated using a quartz crystal oscillator positioned at the sample growth location. During growth, activated oxygen from an electron cyclotron plasma source operating at 40 W and a pressure of 5 × 10^−6^ Torr was used to achieve higher oxidizing power than that achievable with molecular oxygen (O_2_). In addition, all samples were annealed in activated oxygen for 84 s at 650 °C after the growth of each 2 u.c. increment (which also required 84 s to deposit) by programming the source shutters. The samples are designated Sr0, Sr12, Sr25, and Sr50 for *x* values of 0, 0.12, 0.25, and 0.5, respectively. Figure 1b shows the reflection high‐energy electron diffraction (RHEED) patterns for the as‐grown LSNO films. RHEED is a powerful in situ technique used to characterize the surface structure of crystalline materials. These patterns exhibit sharp, bright, unmodulated streaks and no extra diffraction spots, demonstrating excellent crystallinity, smooth surface morphology, and no secondary phases. Atomic force microscope measurements further confirmed flat surfaces with root‐mean square roughness ≈0.2 nm for these LSNO films (Figure S2, Supporting Information). Figure 1c shows crystal truncation rod maps from high‐energy X‐ray surface diffraction.^24^ Bragg spots for the films are aligned with and overlap the substrates peaks, indicating coherent epitaxial growth with the same crystal orientation as the LAO. Keissig fringes are clearly seen along with the main Bragg diffraction rods for all films, demonstrating a well‐defined lattice spacing with sharp interfaces and flat surfaces. Only an a^−^a^−^a^−^ type octahedral rotation pattern, propagated from the substrate, is observed.^25^ No extra a^+^ or b^+^ type rotation is seen, even for the highest *x* values. These results demonstrate that phase pure LSNO of high structural quality grow without secondary phase formation. Figure 1d shows resistivity versus temperature for the LSNO film series. All samples exhibit metallic behavior over the entire temperature range measured.

**Figure 1:**
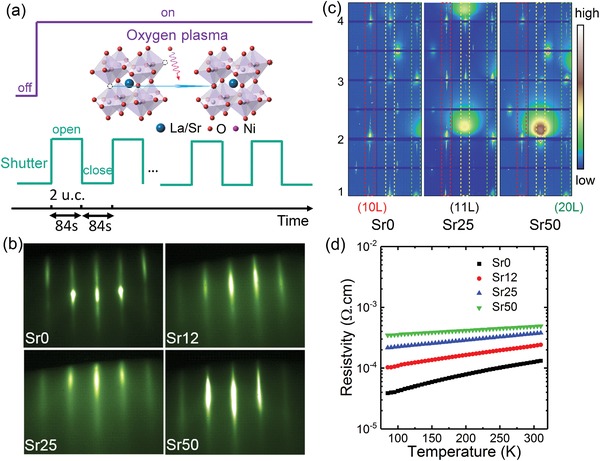
a) Schematic illustration of the controlled growth of LSNO films by OPA‐MBE. Every 2 u.c. deposition (shutter open) was followed by an 84 s in situ annealing (shutter close) in activated oxygen. The inset schematic diagram illustrates that the oxygen vacancies can be healed by the oxygen annealing. b) RHEED patterns for LSNO films grown on LAO viewed along the [100] zone axis. LaNiO_3_ is referred to as “Sr0,” La_0.88_Sr_0.12_NiO_3_ as “Sr12,” La_0.75_Sr_0.25_NiO_3_ as “Sr25,” and La_0.5_Sr_0.5_NiO_3_ as “Sr50.” c) Reciprocal space mapping for the crystal truncation rods of LSNO films. The broad spot comes from parasitic scattering of W stripes used to block the extremely strong substrate Bragg peaks. d) Resistivity versus temperature on warming for LSNO films on LAO substrates.

We now consider the effect of Sr substitution for La on OER activity. LSNO films were electrically contacted on the front and OER activity was measured using a standard three‐electrode configuration in O_2_ saturated 0.1 m potassium hydroxide (KOH) solution.^14^ **Figure** **2**a and Figure S3 (Supporting Information) show cyclic voltammetry (CV) curves to assess OER activity and reveal a clear trend toward enhanced activity with increasing *x*. The specific activities (current density at 1.6 V vs the reversible hydrogen electrode (RHE)) are presented in Figure 2b. It is clear that Sr^2+^ substitution for La^3+^ is favorable for OER, with Sr50 exhibiting 315 µA cm^−2^_oxide_, five higher than that for Sr0 (61 µA cm^−2^_oxide_). For further quantitative comparison, we define the potential required to reach a current density of 50 µA cm^−2^_oxide_, as the OER onset potential (*E*_OER_),^3^, ^17^ and calculate the overpotential (η) according to the formula η = *E*_OER_ − 1.23 V. We summarize the η values for our LSNO films as well as those from other previous studies of pure LNO using the same metric in Figure 2c. For pure LNO, our Sr0 sample shows a η value of ≈0.36 V, similar to those from previous studies.^2^, ^3^ As *x* increases from 0 to 0.5, η continuously decreases, and Sr50 exhibits the smallest η value at 0.29 V, comparable to that of the state‐of‐the‐art catalyst Ba_0.5_Sr_0.5_Co_0.8_Fe_0.2_O_3−_ *_δ_*.^3^ The Tafel plots for our LSNO films shown in Figure 2d display a clear decrease in Tafel slope for higher *x*. The larger increase in current density for a given change in overpotential reveals favorable kinetics and efficient charge transfer at the higher *x* values. Previous consideration of LNO employing Marcus theory with experimentally determined electron affinity and hydroxide affinity have shown that the potentially limiting —O to —OOH step proceeds through a decoupled electron‐then‐proton transfer process,^5^ which coupled with the present decrease in Tafel slopes with Sr suggest that electron transfer is not limiting in the LSNO system. Moreover, Figure 2a shows that the addition of Sr causes an increase in the current associated with the oxidation (1.4 V) and reduction (1.3 V) of nickel. Note that while the current from CV is similar in each cycle for low Sr content, activity increases with cycling for higher Sr content (Figure S3, Supporting Information). For better understanding of this trend, more investigation in our labs by ambient pressure X‐ray photoelectron spectroscopy to probe the surface chemistry in situ and by extensive OER testing under different pH environments are still ongoing.

**Figure 2:**
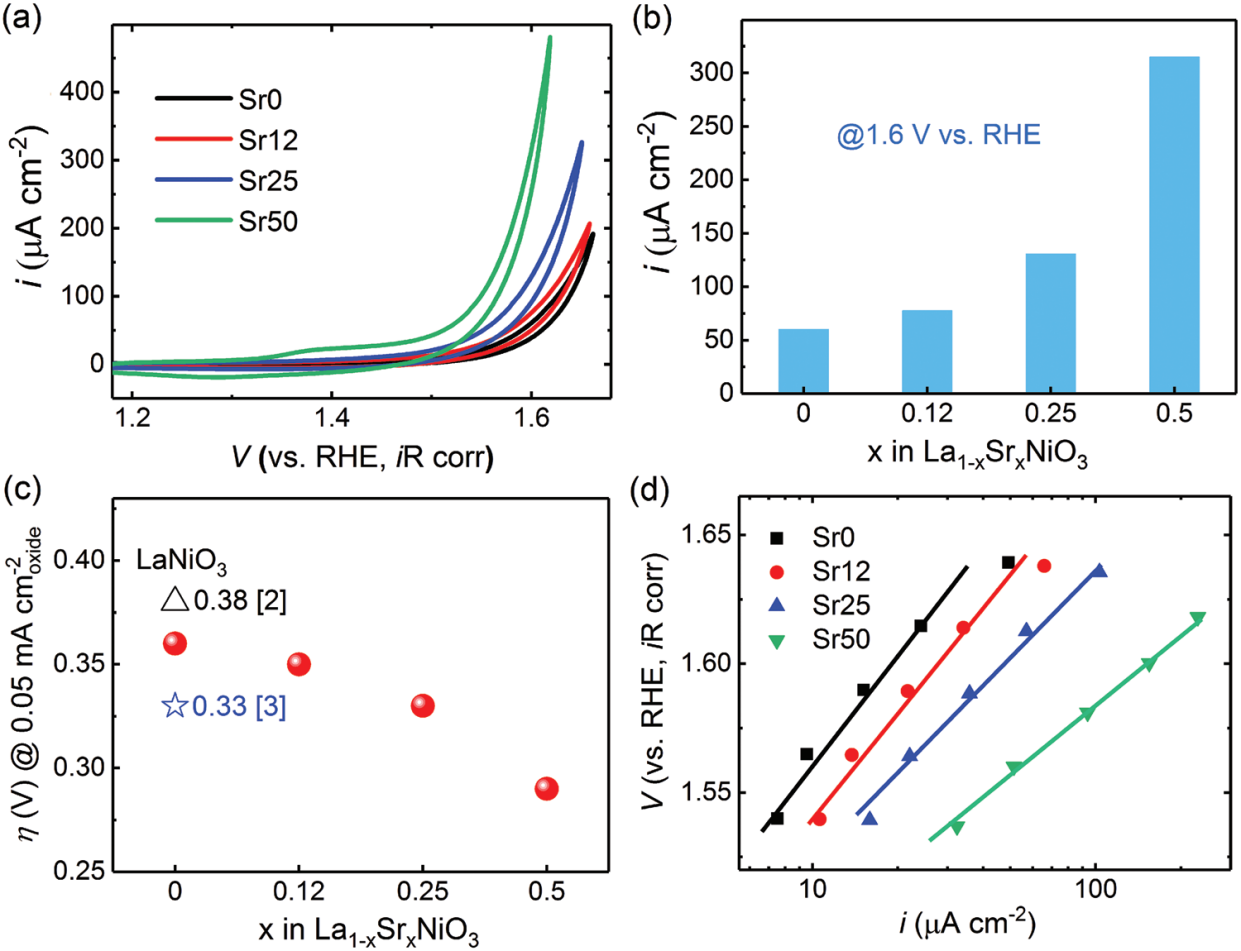
a) Cyclic voltammetry (CV) of LSNO films measured in O_2_ saturated 0.1 m KOH at a scan rate of 10 mV s^−1^, normalized by the specific area with voltage corrected for the electrolyte resistance. b) A comparison of the specific OER activities (current density at 1.6 V vs RHE) of LSNO films. c) Overpotentials (η) for LSNO films as required to obtain 0.05 mA cm^−2^_oxide_ in CV measurements. The η values of LaNiO_3_ reported from the literature are also plotted for comparison. The numbers in brackets are the citation numbers. d) Tafel plot of the OER activities of LSNO films. Points are obtained from steady state measurements by chronoamperometry, with lines as guides to the eye.

As shown in Figure 1d, LNO (Sr0) shows the most conductive behavior. However, Sr0 is not the most OER active. This result is in contrast to previous studies of La_1−_ *_x_*Sr*_x_*CoO_3_, where OER activity increased with conductivity.^19^ While the parent LaNiO_3_ is metallic, LaCoO_3_ is insulating. Conductivity is proportional to the product of mobility and carrier concentration. For the La_1−_ *_x_*Sr*_x_*CoO_3_ system, Sr doping increases the carrier concentration, leading to an increase in conductivity. But for La_1−_ *_x_*Sr*_x_*NiO_3_, Sr doping may increase the carrier density, but it will also decrease the mobility due to the increase of ionized impurity scattering. The product of these two effects induces a decrease in conductivity. In order to better understand the relationship between OER activity and electronic structure, we performed XAS and XPS measurements at room temperature to probe the unoccupied and occupied electronic states, respectively. **Figure** **3**a and Figure S4 (Supporting Information) show the normalized O K‐edge XAS pre‐edge features for the film series. The peaks fall at ≈528 eV, and correspond to a transition from the O 1s core to the lowest unoccupied hybridized O 2p–Ni 3d band. We designate this transition as 3d^8^ L → c3d^8^ where L and c denote a hole in the O 2p ligand orbital and an O 1s core hole, respectively.^26^, ^27^, ^28^ The O K prepeak width can be used to measure the degree of O 2p–Ni 3d hybridization as it is related to the Ni—O—Ni bond angle, as has been well elucidated in previous studies.^29^, ^30^, ^31^ Figure 3a,b shows that the O K pre‐edge peak width increases with increasing *x*, suggesting an increase in Ni 3d–O 2p hybridization. According to previous studies,^3^, ^32^ the OER activity is strongly related to the ability of the electrocatalyst surface to bind oxygen. The reaction intermediates, including *OH, *O, *OOH, interact with the catalyst surface through the oxygen atom.^33^ Enhanced Ni 3d–O 2p hybridization can improve electron extraction from oxygen adsorbates, thus increasing the OER activity.^11^ Note that this enhancement of Ni 3d–O 2p hybridization may result from the increase of Ni oxidation state by Sr^2+^ substitution. Our in situ XPS measurements (Figure 3c and Figure S5 (Supporting Information)) indeed reveal a shift of the Ni 2p_1/2_ peak to the higher binding energy with increasing *x*, suggesting an increase in Ni valence. While the elusive Ni^4+^ is difficult to pinpoint spectroscopically, the X‐ray diffraction (XRD) data suggest its existence as follows. Sr^2+^ has a larger ionic radius than La^3+^ (1.26 vs 1.16 Å),^34^ thus the lattice parameters of LSNO should increase as *x* increases. However, as shown in Figure 1c and Figure S6 (Supporting Information), the lattice parameter becomes smaller with Sr doping, which can only be rationalized by Ni^4+^ creation. The smaller ionic radius of Ni^4+^ would shorten the Ni—O bond length, leading to the enhancement of covalency or Ni 3d–O 2p hybridization.

**Figure 3:**
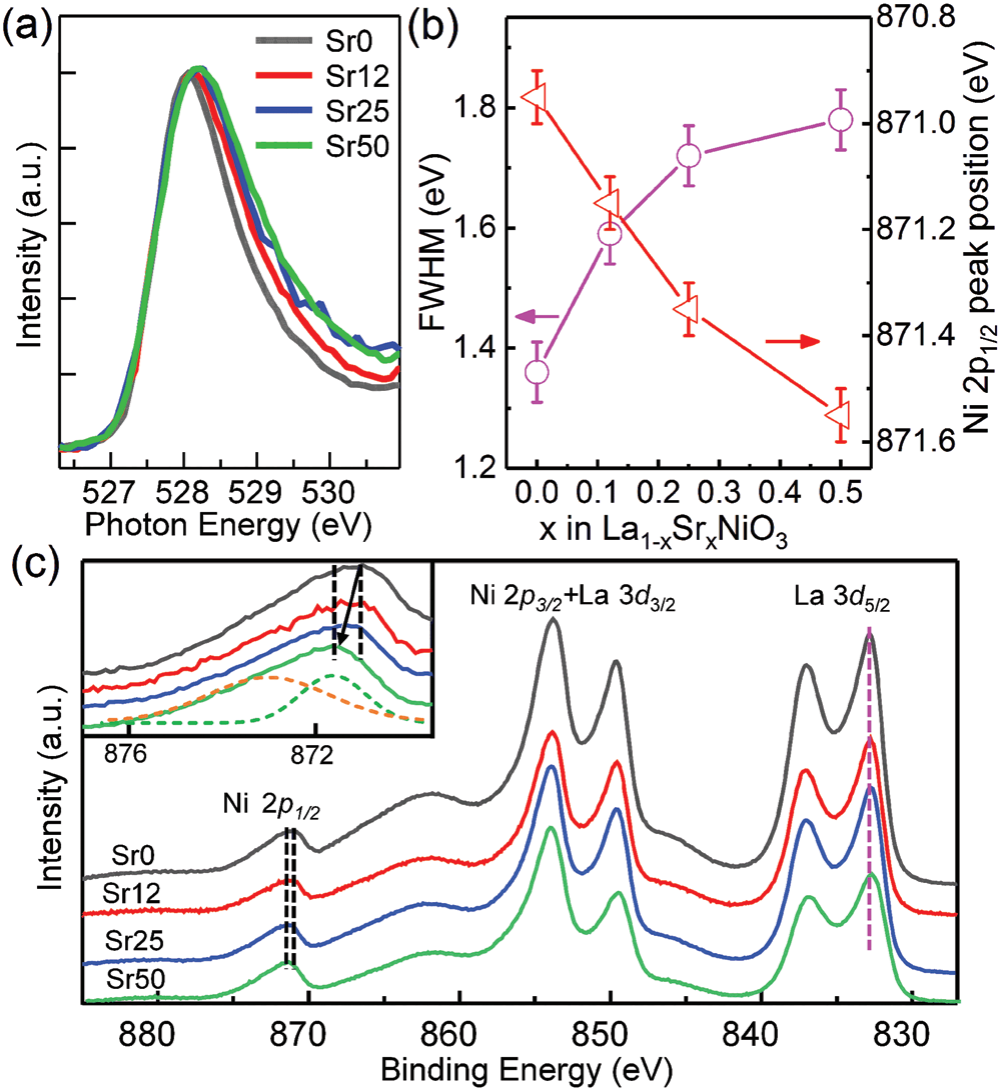
a) Soft X‐ray absorption O K pre‐edge spectra for LSNO films taken at 300 K. b) O K prepeak full width at half maximum (FWHM) values near 528 eV and Ni 2p_1/2_ peak energies extracted from (c) versus *x*. c) La 3d and Ni 2p XPS measured in situ for the LSNO film series. All spectra are shifted so the associated O 1s peaks fall at 530.0 eV. As a guide to the eye, we mark the La 3d_5/2_ peak position with a magenta dashed line. Inset: Ni 2p_1/2_ spectra for which the peak shift with *x* is clearly seen. These asymmetric peaks were fit to a pair of model functions (dashed curves) solely for the purpose of tracking changes in the binding energy with *x*, as plotted in panel (b).

The enhancement in hybridization is also reflected in the evolution of the valence band (VB). A clear upward shift of the O 2p band is observed in our XPS measurements (**Figure** **4**a) and DFT calculations (Figure 4b). For pure LNO (Sr0), the VB spectrum consists of three features labeled as A, B, and C. By contrast, Sr50 exhibits only two distinct components as features A and B merge. According to our DFT calculations (Figure 4b) and previous reports,^35^, ^36^ feature A is assigned to a predominantly occupied Ni 3d band with minor O 2p character. Feature B arises primarily from O—O interactions and is assigned to the nonbonding O 2p band with only a small covalent admixture of Ni 3d, whereas feature C is assigned to bonding states. The experimental spectra are in good agreement with our DFT calculations; the O 2p band shifts upward in energy and moves close to *E*_F_, inducing mixing of features A and B, as observed in the VB spectra for Sr50 (Figure 4a). The merging of features A and B is due not only to the reduced spacing between the two peaks, but also to the fact the antibonding state has more O 2p character than the nonbonding state with enhanced Ni 3d–O 2p hybridization (Figure 4b). Figure 4c shows the computed average onsite energies of the O 2p and Ni 3d bands relative to *E*_F_. With increasing *x*, O 2p bands shift up and move closer to *E*_F_. The upshift of the O 2p bands, which enhances Ni 3d–O 2p hybridization (covalency), is due to the decrease in Coulomb interaction with the A site cation when the valence of the latter changes from 3+ to 2+. Viewed from a different perspective, Sr^2+^ substitution for La^3+^ results in hole doping in LNO, driving a downward shift in *E*_F_ relative to pure LNO. As the O 2p band center shifts upward and approaches *E*_F_, the antibonding states below *E*_F_ exhibit greater oxygen character, which in turn promotes electron transfer to and from oxygen by allowing Ni 3d to mix more strongly with O 2p, thus leading to higher OER activity. We note that a shift of the O 2p band closer to the Fermi level leads to activation during cycling in Co oxides^12^ and the formation of an amorphous Co oxide layer,^37^ and while LNO's O 2p band center is low enough such change is unexpected, the increase with Sr content could trigger such a structural change during OER. However, changes in LSNO film capacitance with cycling was not observed, suggesting that the electrochemical surface area remains unchanged with cycling.

**Figure 4:**
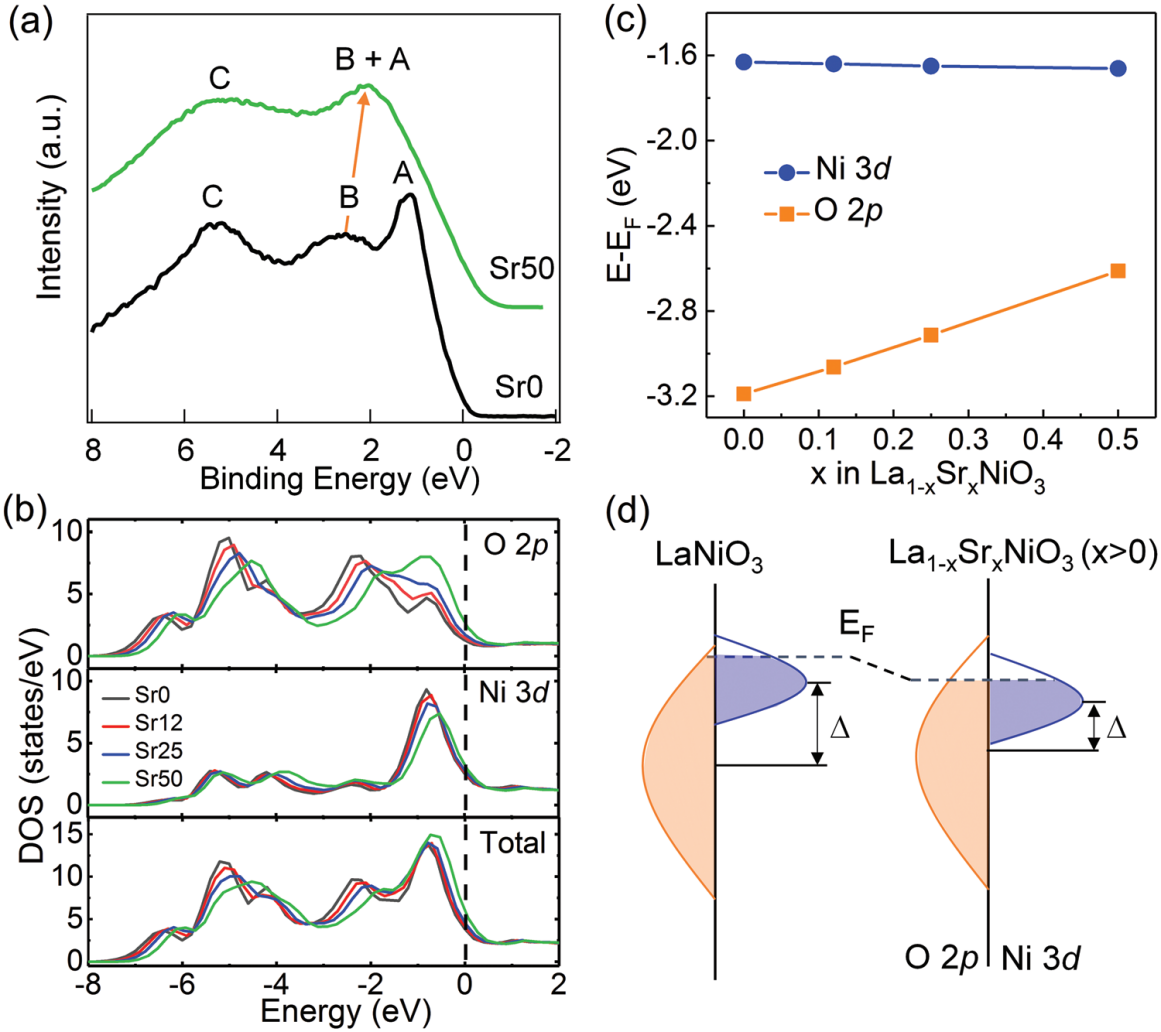
a) XPS VB spectra for Sr0 and Sr50. b) Computed partial and total densities of states from DFT. The black dashed line denotes *E*_F_. c) Average onsite energies of O 2p and Ni 3d orbitals with *E*_F_ as the reference. d) Schematic energy band diagram for LaNiO_3_ and La_1−_ *_x_*Sr*_x_*NiO_3_ (*x* > 0), where *Δ* is the charge transfer energy.

In perovskite transition metal oxides, the extent of metal 3d–oxygen 2p hybridization scales with the ratio of the transfer integral to charge transfer energy (*∆*).^38^ For nickelates, the Ni 3d–O 2p hybridization is primarily controlled by *∆*, while the transfer integral has a weaker influence.^36^ The evolution of Ni 3d and O 2p bands revealed in our VB measurements and DFT calculations confirm the decrease in *∆* with increasing *x* as a result of stronger hybridization. Based on the above analysis, we show a schematic energy band diagram (Figure 4d) for pure LNO and LSNO (*x* > 0). The decrease in *∆* should enhance OER activity as *x* increases. Hole doping of the O 2p band can lead to more effective electronic screening at the surface, decreasing the barrier for the electron transfer step of the OER.^5^

In summary, we have demonstrated that Sr^2+^ substitution for La^3+^ in LaNiO_3_ is an effective way to improve OER activity. The combination of experimental results with DFT calculations demonstrates that synthesizing this solid solution reduces the charge transfer energy and strengthens Ni 3d–O 2p hybridization, which in turn leads to higher OER activity. At *x* = 0.5 (La_0.5_Sr_0.5_NiO_3_), the overpotential required to achieve 50 µA cm^−2^_oxide_ is 0.29 V, which is comparable to that of the state‐of‐the‐art catalyst Ba_0.5_Sr_0.5_Co_0.8_Fe_0.2_O_3−_ *_δ_*. Our study offers new insights into the effect of solid solution formation on OER activity in perovskite nickelates and will promote the application of this solid‐state chemistry to make effective oxygen electrocatalysts.

## Conflict of Interest

The authors declare no conflict of interest.

## Supporting information

Supplementary

## References

[1] Z. W. Seh , J. Kibsgaard , C. F. Dickens , I. Chorkendorff , J. K. Norskov , T. F. Jaramillo , Science 2017, 355, eaad4998. 28082532 10.1126/science.aad4998

[2] J. R. Petrie , V. R. Cooper , J. W. Freeland , T. L. Meyer , Z. Zhang , D. A. Lutterman , H. N. Lee , J. Am. Chem. Soc. 2016, 138, 2488. 26866808 10.1021/jacs.5b11713

[3] J. Suntivich , K. J. May , H. A. Gasteiger , J. B. Goodenough , Y. Shao‐Horn , Science 2011, 334, 1383. 22033519 10.1126/science.1212858

[4] J. Hwang , R. R. Rao , L. Giordano , Y. Katayama , Y. Yu , Y. Shao‐Horn , Science 2017, 358, 751. 29123062 10.1126/science.aam7092

[5] W. T. Hong , K. A. Stoerzinger , Y.‐L. Lee , L. Giordano , A. Grimaud , A. M. Johnson , J. Hwang , E. J. Crumlin , W. Yang , Y. Shao‐Horn , Energy Environ. Sci. 2017, 10, 2190.

[6] J. T. Mefford , X. Rong , A. M. Abakumov , W. G. Hardin , S. Dai , A. M. Kolpak , K. P. Johnston , K. J. Stevenson , Nat. Commun. 2016, 7, 11053. 27006166 10.1038/ncomms11053 PMC4814573

[7] X. Cheng , E. Fabbri , M. Nachtegaal , I. E. Castelli , M. El Kazzi , R. Haumont , N. Marzari , T. J. Schmidt , Chem. Mater. 2015, 27, 7662.

[8] Y. Tong , Y. Guo , P. Chen , H. Liu , M. Zhang , L. Zhang , W. Yan , W. Chu , C. Wu , Y. Xie , Chem 2017, 3, 812.

[9] F. Calle‐Vallejo , N. G. Inoglu , H.‐Y. Su , J. I. Martínez , I. C. Man , M. T. Koper , J. R. Kitchin , J. Rossmeisl , Chem. Sci. 2013, 4, 1245.

[10] C. Wei , Z. Feng , G. G. Scherer , J. Barber , Y. Shao‐Horn , Z. J. Xu , Adv. Mater. 2017, 29, 1606800. 10.1002/adma.201606800 28394440

[11] J. Suntivich , W. T. Hong , Y.‐L. Lee , J. M. Rondinelli , W. Yang , J. B. Goodenough , B. Dabrowski , J. W. Freeland , Y. Shao‐Horn , J. Phys. Chem. C 2014, 118, 1856.

[12] A. Grimaud , K. J. May , C. E. Carlton , Y.‐L. Lee , M. Risch , W. T. Hong , J. Zhou , Y. Shao‐Horn , Nat. Commun. 2013, 4, 2439. 24042731 10.1038/ncomms3439

[13] S. A. Akhade , J. R. Kitchin , J. Chem. Phys. 2012, 137, 084703. 22938255 10.1063/1.4746117

[14] L. Wang , K. A. Stoerzinger , L. Chang , J. Zhao , Y. Li , C. S. Tang , X. Yin , M. E. Bowden , Z. Yang , H. Guo , Adv. Funct. Mater. 2018, 28, 1803712.

[15] L. Wang , K. A. Stoerzinger , L. Chang , X. Yin , Y. Li , C. S. Tang , E. Jia , M. E. Bowden , Z. Yang , A. Abdelsamie , ACS Appl. Mater. Interfaces 2019, 11, 12941. 30834739 10.1021/acsami.8b21301

[16] J. Bak , H. B. Bae , J. Kim , J. Oh , S.‐Y. Chung , Nano Lett. 2017, 17, 3126. 28394129 10.1021/acs.nanolett.7b00561

[17] S. Yagi , I. Yamada , H. Tsukasaki , A. Seno , M. Murakami , H. Fujii , H. Chen , N. Umezawa , H. Abe , N. Nishiyama , Nat. Commun. 2015, 6, 8249. 26354832 10.1038/ncomms9249 PMC4579779

[18] S. She , J. Yu , W. Tang , Y. Zhu , Y. Chen , J. Sunarso , W. Zhou , Z. Shao , ACS Appl. Mater. Interfaces 2018, 10, 11715. 29546981 10.1021/acsami.8b00682

[19] K. A. Stoerzinger , X. R. Wang , J. Hwang , R. R. Rao , W. T. Hong , C. Rouleau , D. Lee , Y. Yu , E. J. Crumlin , Y. Shao‐Horn , Top. Catal. 2018, 61, 2161.

[20] R. Sankannavar , K. Sandeep , S. Kamath , A. K. Suresh , A. Sarkar , J. Electrochem. Soc. 2018, 165, J3236.

[21] R. Comes , S. Chambers , Phys. Rev. Lett. 2016, 117, 226802. 27925724 10.1103/PhysRevLett.117.226802

[22] L. Wang , Y. Du , L. Chang , K. Stoerzinger , M. Bowden , J. Wang , S. Chambers , Appl. Phys. Lett. 2018, 112, 261601.

[23] A. S. Disa , D. Kumah , J. Ngai , E. D. Specht , D. Arena , F. J. Walker , C. H. Ahn , APL Mater. 2013, 1, 032110.

[24] J. Gustafson , M. Shipilin , C. Zhang , A. Stierle , U. Hejral , U. Ruett , O. Gutowski , P.‐A. Carlsson , M. Skoglundh , E. Lundgren , Science 2014, 343, 758. 24482118 10.1126/science.1246834

[25] S. J. May , J. W. Kim , J. M. Rondinelli , E. Karapetrova , N. A. Spaldin , A. Bhattacharya , P. J. Ryan , Phys. Rev. B 2010, 82, 014110.

[26] P. Kuiper , G. Kruizinga , J. Ghijsen , G. Sawatzky , H. Verweij , Phys. Rev. Lett. 1989, 62, 221. 10039954 10.1103/PhysRevLett.62.221

[27] M. Abbate , G. Zampieri , F. Prado , A. Caneiro , J. Gonzalez‐Calbet , M. Vallet‐Regi , Phys. Rev. B 2002, 65, 155101.

[28] N. Palina , L. Wang , S. Dash , X. Yu , M. B. Breese , J. Wang , A. Rusydi , Nanoscale 2017, 9, 6094. 28447095 10.1039/c7nr00742f

[29] S. Middey , D. Meyers , S. K. Ojha , M. Kareev , X. Liu , Y. Cao , J. Freeland , J. Chakhalian , Phys. Rev. B 2018, 98, 045115. 10.1103/PhysRevLett.120.156801 29756872

[30] J. Liu , M. Kargarian , M. Kareev , B. Gray , P. J. Ryan , A. Cruz , N. Tahir , Y.‐D. Chuang , J. Guo , J. M. Rondinelli , Nat. Commun. 2013, 4, 2714. 24193317 10.1038/ncomms3714

[31] J. Chakhalian , J. Rondinelli , J. Liu , B. Gray , M. Kareev , E. Moon , N. Prasai , J. Cohn , M. Varela , I.‐C. Tung , Phys. Rev. Lett. 2011, 107, 116805. 22026694 10.1103/PhysRevLett.107.116805

[32] D.‐Y. Kuo , C. J. Eom , J. K. Kawasaki , G. Petretto , J. N. Nelson , G. Hautier , E. J. Crumlin , K. M. Shen , D. G. Schlom , J. Suntivich , J. Phys. Chem. C 2018, 122, 4359.

[33] I. C. Man , H. Y. Su , F. Calle‐Vallejo , H. A. Hansen , J. I. Martínez , N. G. Inoglu , J. Kitchin , T. F. Jaramillo , J. K. Nørskov , J. Rossmeisl , ChemCatChem 2011, 3, 1159.

[34] R. D. Shannon , Acta Crystallogr., Sect. A: Found. Adv. 1976, 32, 751.

[35] D. Sarma , N. Shanthi , P. Mahadevan , J. Phys.: Condens. Matter 1994, 6, 10467.

[36] S. Barman , A. Chainani , D. Sarma , Phys. Rev. B 1994, 49, 8475. 10.1103/physrevb.49.8475 10009616

[37] M. Risch , A. Grimaud , K. J May , K. A Stoerzinger , T. J Chen , A. N Mansour , Y. Shao‐Horn , J. Phys. Chem. C 2013, 117, 8628.

[38] M. N. Grisolia , J. Varignon , G. Sanchez‐Santolino , A. Arora , S. Valencia , M. Varela , R. Abrudan , E. Weschke , E. Schierle , J. E. Rault , J. P. Rueff , A. Barthelemy , J. Santamaria , M. Bibes , Nat. Phys. 2016, 12, 484. 27158255 10.1038/nphys3627 PMC4856211

