# Supplementary material for: Tuning the Electronic Structure of LaNiO3 through Alloying with Strontium to Enhance Oxygen Evolution Activity
Source: Adv Sci (Weinh). 2019 Aug 7;6(19):1901073. doi: 10.1002/advs.201901073 (PMC6774028; doi:10.1002/advs.201901073)
Supplement: Supplementary file 1 — Supplementary [file ADVS-6-1901073-s001.pdf]

Copyright WILEY-VCH Verlag GmbH & Co. KGaA, 69469 Weinheim, Germany, 2019.

ADVANCED SCIENCE
Open Access

Supporting Information

for *Adv. Sci.,* DOI: 10.1002/advs.201901073

Tuning the Electronic Structure of $LaNiO_3$ through Alloying with Strontium to Enhance Oxygen Evolution Activity

Jishan Liu, Endong Jia, Le Wang, Kelsey A. Stoerzinger,* Hua Zhou, Chi Sin Tang, Xinmao Yin, Xu He, Eric Bousquet, Mark E. Bowden, Andrew T. S. Wee, Scott A. Chambers, and Yingge Du**

Supporting information

Tuning Electronic Structure of $LaNiO_3$ through Strontium Doping to Enhance Oxygen Evolution Reaction Activity

Jishan Liu, Endong Jia, Le Wang, Kelsey A. Stoerzinger*, Hua Zhou, Chi Sin Tang, Xinmao Yin, Xu He, Eric Bousquet, Mark E. Bowden, Andrew Wee, Scott A. Chambers, and Yingge Du**

E-mail: le.wang@pnnl.gov, kelsey.stoerzinger@oregonstate.edu, yingge.du@pnnl.gov

Experimental details

Thin Film Growth: High-quality epitaxial $La_{1-x}Sr_xNiO_3$ films with x = 0, 0.12, 0.25, 0.5, 0.75 and 1.0 and thickness of 20 u.c. were grown on (001)-oriented LAO substrates by OPA-MBE. La, Sr, and Ni were evaporated from Knudsen effusion cells, and evaporation rates were calibrated using a quartz crystal oscillator. The substrate temperature was set to 650 °C, and the activated oxygen partial pressure was kept at ~5×10^{-6} Torr during growth. *In situ* reflection high-energy electron diffraction (RHEED) was used to monitor the overall growth rate, and surface crystallography and structure. A growth and annealing sequence controlled by shutter was used to heal the oxygen vacancies by soaking 2 u.c. film increments in activated oxygen between growth depositions. After growth, the activated oxygen partial pressure was raised to 3×10^{-5} Torr for an additional 30 mins to further eliminate oxygen vacancies. The sample was then cooled to room temperature at 5 °C /min.

XPS and XAS Measurements: After growth, the films were transferred to an appended ultrahigh vacuum chamber equipped with a VG/Scienta R3000 electron energy analyzer and a monochromatic Al-Kα x-ray source for high-resolution XPS studies. XAS measurements

were also performed at the SINS beamline at the Singapore Synchrotron Light Source (SSLS). The XAS spectra were measured in total electron yield (TEY) mode by collecting the sample current with a photon energy resolution of 200 meV.

Structure Characterization: Atomic force microscope (AFM) was used to examine film surface morphology. The root-mean surface roughness is ~ 0.2 nm for all LSNO films, as shown in Figure S2. XRD measurements were performed using a Rigaku SmartLab instrument. The crystal truncation rod maps of these LSNO films were investigated at the 1-ID beamline of Advanced Photon Source.

Electrical Measurements: In-plane transport properties of LSNO films were investigated using a Hall measurement system in the temperature range 85−310 K. Measurements were done in the van der Pauw geometry with square samples (~5×5 mm^2) and silver paste in the corners.

Electrochemical Experiments: The experimental setups for OER measurements have been described elsewhere.[S1] To summarize, electrical contact was made to the front of the LSNO film (rather than the insulating LAO substrate) using GaIn eutectic (Sigma, >99.99%) and Ag paste (Liebsilber, Ted Pella). The Cu wire contact, back and sides of the electrode were covered with inert epoxy (Locite 9460). The sample was ~1 cm from the Pt wire counter electrode (ALS Co.) and ~1 cm from the saturated calomel reference electrode (RE-2BP, ALS Co.) reference electrode (ALS Co.), which was calibrated to the RHE in the same electrolyte. The 0.1 M KOH (Sigma Aldrich, 99.99%) electrolyte was prepared with Nanopure water (18.2 MΩ-cm). The ~15 mL volume was stirred and bubbled with ultra-high purity O_2 gas before and during data collection to fix the potential of oxygen redox. Measurements were conducted with a Biologic SP-200 potentiostat at 10 mV/s, and voltages corrected for the

electrolyte/cell resistance from the high frequency intercept of the real impedance (V-iR). For measurements of in-plane voltage drop, 5 mM each of $K_3Fe(CN)_6$ and $K_4Fe(CN)_6*3H_2O$ (Sigma, >99%) were added to the 0.1 M KOH electrolyte, and the electrolyte saturated with ultra-high purity N_2 in a glass cell with a Ag/AgCl reference electrode (ALS Co.) calibrated to the RHE. As AFM measurements confirmed flat surfaces with root-mean square roughness ~0.2 nm for these films, the OER activity was normalized to the film surface area exposed to the electrolyte, digitally analyzed with ImageJ software.[S2] We refer to the activity normalized by measure as intrinsic activity, in units of $\mu A/cm^2_{oxide}$. For reference, the exposed surface area of the catalyst was on the order of ~0.4 cm^2_{oxide}, and with the 20 u.c. thickness this corresponds to a nominal mass loading of 5.3 $\mu g/cm^2$ for a total catalyst loading of ~2 μg. The corresponding mass activities from Figure 2b then range from 11.5 A/g for Sr0 to 59.7 A/g for Sr50 at 1.6 V vs. RHE. In addition, the electrochemical surface area (ECSA) was determined by comparing the capacitance measured from 1.16-1.26 V vs. RHE, over which no redox features are observed, at 10 mV/s in N_2-saturated KOH, taking the average of the cathodic and anodic charge. Sr = 0, 12.5, and 25 have comparable capacitance, and thus similar ECSA assuming a constant intrinsic capacitance of 0.04 mF/cm^2. In contrast, the capacitance and ECSA of Sr = 50 is ~1.5x higher. This is comparable to the differences in current obtained from the ferri/ferrocyanide reaction in a quiescent setup at 1.52 V vs RHE. Regardless of ambiguity due to the unknown intrinsic capacitances of these surfaces,[S3] the trends in OER activity increasing with Sr are unchanged by normalizing to the calculated ECSA rather than the surface area determined by ImageJ. We note that this calculated ECSA exceeds the total area of the as-deposited film prior to covering a portion of the film with the electrical contact (not exposed to the electrolyte), which is in contrast to the AFM root mean square roughness of ~0.2 nm.

Table S1. Comparison of surface areas calculated by ImageJ analysis of electrode images and the electrochemical surface area (ECSA) calculated assuming an intrinsic capacitance for all electrodes of 0.04 mF/cm^2.

Samples	ImageJ (cm^2)	Average capacitance (mF)	ECSA using 0.04 mF/cm^2 (cm^2)	Roughness factor
Sr0	0.14	0.018	0.441	3.150
Sr12	0.159	0.019	0.482	3.034
Sr25	0.122	0.016	0.399	3.273
Sr50	0.133	0.027	0.682	5.126

DFT calculation: Our DFT calculation were carried out with VASP. A plane wave basis set with cutoff energy of 500 eV, PBEsol functional and 6*6*6 Gamma-centered k-mesh were used. We used the virtual crystal approximation (VCA) to simulate solid solution formation. We then used Wannier90 to constructure the maximally localized Wannier functions (MLWF), in order to map the Kohn-Sham Hamiltonian to an effective Hamiltonian with a local basis set of O 2*p* and Ni 3*d* orbitals. From that Hamiltonian, we determined the on-site energy average of O 2*p* and Ni 3*d* states.

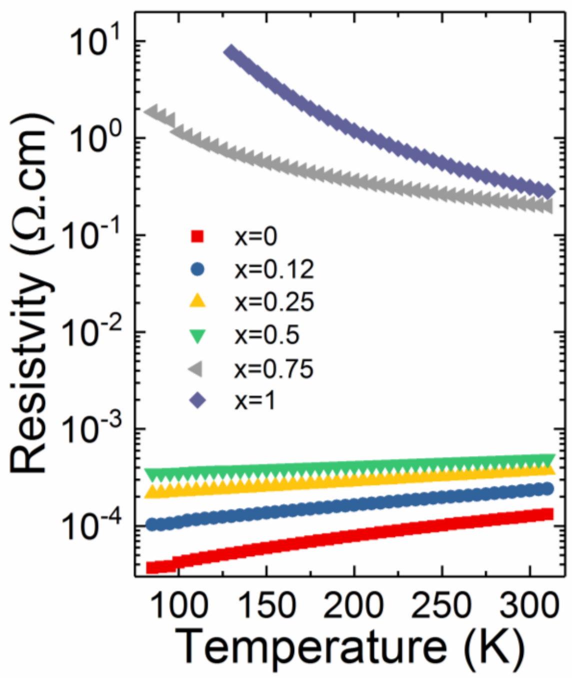

Figure S1. Resistivity vs. temperature curves on warming for $La_{1-x}Sr_xNiO_3$ ($0 \leq x \leq 1$) films on LAO substrates.

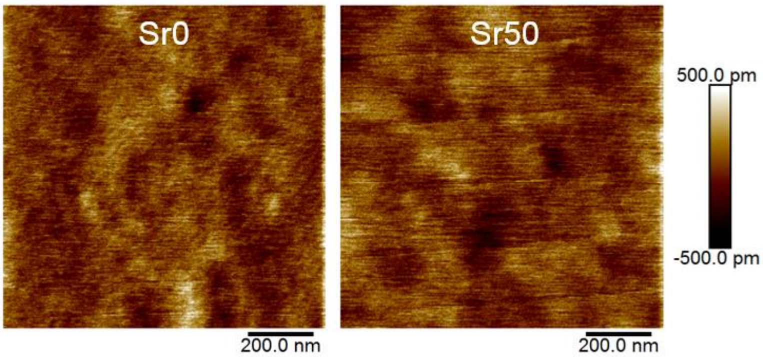

Figure S2. Atomic force microscopy images of SrO and Sr50 films, respectively, both of which show a flat surface with a root-mean square roughness <0.2 nm.

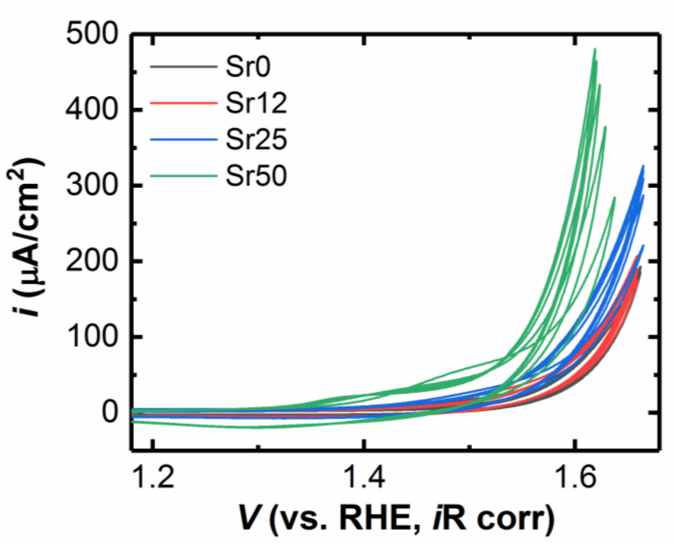

Figure S3. Five cycles of cyclic voltammetry (CV) of LSNO films, where the OER is measured at a scan rate of 10 mV s^{-1} in O_2-saturated 0.1 M KOH. The activity remains similar over 5 cycles for low Sr content, but increases with cycling for Sr50. This activation is the study of ongoing work in our laboratory.

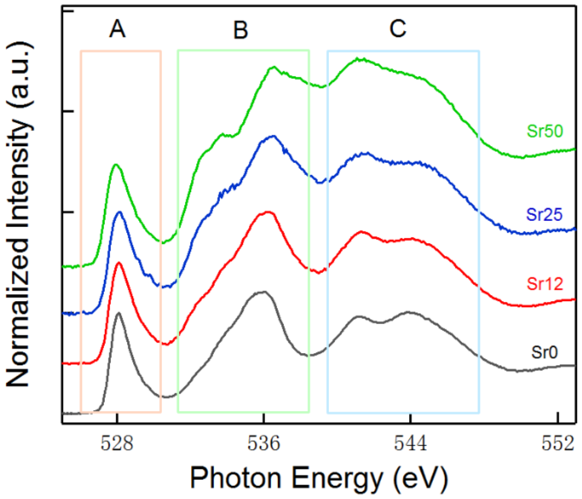

Figure S4. X-ray absorption spectroscopy at the oxygen K edge for LSNO (x=0, 0.12, 0.25, 0.5) films grown on LAO. The highlighted region A is assigned to the O 2*p*-Ni 3*d* hybridized state, B and C are from the excitations of O 1*s* to O 2*p*-La(Sr) state and O 2*p*-Ni 4*sp*, respectively.[S4,S5]

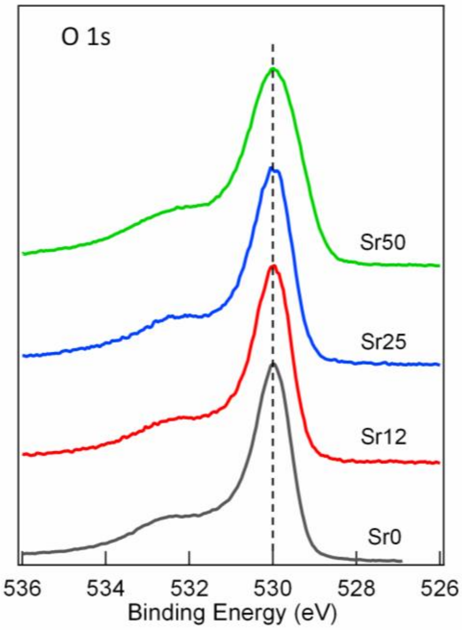

Figure S5. *In-situ* O 1s XPS spectra of LSNO samples with different Sr content. These as-grown LSNO films show a main peak (~530 eV) associated with the lattice oxygen, which displays broadening with increasing Sr content.

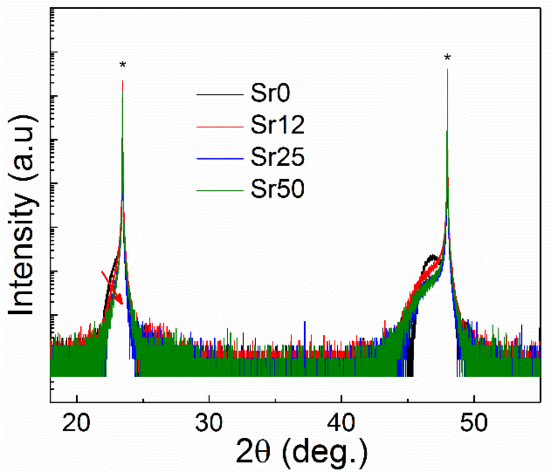

Figure S6. Extended XRD θ-2θ patterns for LSNO films grown on LAO(001) substrates, from which the single-phase LSNO films with c-axis orientation can be deduced. The substrate (001) and (002) diffraction peaks are displayed as stars. The red arrow shows that the (001) diffraction peaks of these LSNO films shift to higher angle with Sr doping, indicating the c-axis lattice parameter decreases with Sr doping.

References

[S1] L. Wang, K. A. Stoerzinger, L. Chang, J. Zhao, Y. Li, C. S. Tang, X. Yin, M. E. Bowden, Z. Yang, H. Guo, *Adv. Funct. Mater*. **2018**, *28*, 1803712.

[S2] C. A. Schneider, W. S. Rasband, K. W. Eliceiri, *Nat. Methods* **2012**, 9 (7), 671.

[S3] C. C. L. McCrory, S. Jung, J. C. Peters, and T. F. Jaramillo, *J. Am. Chem. Soc.* **2013**, 135, 16977.

[S4] J. Suntivich, W. T. Hong, Y.-L. Lee, J. M. Rondinelli, W. Yang, J. B. Goodenough, B. Dabrowski, J. W. Freeland, Y. Shao-Horn, *J. Phys.Chem. C* **2014**, *118*, 1856.

[S5] M. Golalikhani, Q. Lei, R. U. Chandrasena, L. Kasaei, H. Park, J. Bai, P. Orgiani, J. Ciston, G. E. Sterbinsky, D. A. Arena, P. Shafer, E. Arenholz, B. A. Davidson, A. J. Gray, X. X. Xi, Nat. Commun. *Nat. Commun.* **2018**, *9,* 2206.
